# Intra-hospital transport of critically ill patients with rapid response team and risk factors for cardiopulmonary arrest: A retrospective cohort study

**DOI:** 10.1371/journal.pone.0213146

**Published:** 2019-03-05

**Authors:** Hyun Ju Min, Hyung-Jun Kim, Dong Seon Lee, Yun Young Choi, Miae Yoon, Dayoon Lee, Jun Yeun Cho, Jong Sun Park, Young-Jae Cho, Ho Il Yoon, Jae Ho Lee, Choon-Taek Lee, Yeon Joo Lee

**Affiliations:** 1 Division of Pulmonary and Critical Care Medicine, Department of Internal Medicine, Seoul National University Bundang Hospital, Seongnam-si, Gyeonggi-do, Republic of Korea; 2 Department of Nursing, Seoul National University Bundang Hospital, Seongnam, Gyeonggi-do, Republic of Korea; 3 Department of Internal Medicine, Armed Forces Daegu Hospital, Gyeongsan-si, Gyeongsangbuk-do, Republic of Korea; Azienda Ospedaliero Universitaria Careggi, ITALY

## Abstract

**Introduction:**

This study aimed to determine the occurrence rate and risk factors of cardiopulmonary arrest (CPA) during intra-hospital transport (IHT) among critically ill patients, accompanied by a rapid response team (RRT).

**Methods:**

We performed a retrospective cohort study in a 1300-bed tertiary-care teaching hospital. Data of all admitted patients transported within the hospital and accompanied by the RRT from October 2012 to May 2016 were included. We compared patients with CPA (+) and patients without CPA (-) to identify risk factors for CPA during transport.

**Results:**

Among 535 patients, CPA occurred in eight (1.5%) patients during IHT. There were no significant differences in age, sex, and comorbidities between groups. More patients in the CPA (+) group than in the CPA (-) group received manual ventilation during IHT (75% vs. 23.0%, p = 0.001). An increased risk of CPA (p<0.001) corresponded with a higher number of vasopressors used during IHT. In univariate logistic regression analysis, history of myocardial infarction (OR 10.7, 95% CI 2.4–50.5, p = 0.005), manual ventilation (OR 10.1, 95% CI 2.0–50.5, p = 0.005), and use of three or more vasopressors (OR 11.1, 95% CI 2.5–48.9, p = 0.001) were significantly associated with risk of CPA during RRT-led IHT.

**Conclusions:**

Despite accompaniment by a specialized team such as the RRT, CPA can occur during IHT. History of myocardial infarction, manual ventilation with bag-valve mask, and the use of three or more vasopressors were independent risk factors of CPA during IHT of critically ill patients accompanied by the RRT.

## Introduction

In practical guidelines on the transport of critically ill patients, published in 2004 [1], four components of intra-hospital transport (IHT) were suggested as essential for safe patient transport: “pre-transport coordination, accompanying personnel, equipment, and monitoring during transport”. Ideally, these guidelines recommend that all critical care transports be performed by specially trained individuals, as qualified personnel in critical care can adequately cope with at-risk patients and intervene in the event of serious adverse events (AEs), such as hypoxia, hypotension, etc. as well as minor AEs [1]. Nevertheless, even when accompanied by specially trained teams, cardiopulmonary arrest (CPA)—one of the most serious AEs—can occur during IHT despite the care provided by a dedicated transport team [2] or specially trained intensive care unit (ICU) staff [3].

A rapid response team (RRT) was launched in our institution in October 2012. The RRT in our institution began to accompany the transfers of critical patients, in addition to their regular task of early detection and management of “at risk” patients. The RRT staffs have existing competencies in providing care for critically ill patients, and the efficacy of the team was previously shown to decrease the overall rates of CPA among hospitalized patients after RRT implementation [4].

CPA during IHT is the most serious complication that can occur during the transport process and can result in very poor patient outcomes. Family members of the patient can perceive CPA during transport as an accident caused by medical-staff negligence which may result in a lawsuit against the medical staff or institution. Although previous studies have reported a rate of CPA during IHT that ranged from 0.3–3% [5–9], these studies did not evaluate the risk factors for CPA during IHT, instead they focused on the risk factors for the broader range of overall AEs during IHT. Therefore, we aimed to determine the occurrence rate of CPA during IHT among critically ill patients, and to identify risk factors for CPA during transport, accompanied by the RRT comprised of specialized critical care staff.

## Materials and methods

### Study design

We performed a retrospective, single center cohort study using the RRT registry data of a 1300-bed tertiary-care teaching hospital affiliated with Seoul National University in South Korea. All IHTs of admitted patients accompanied by the RRT from October 2012 to May 2016 were included in this study. We extracted the transport-monitoring list from the RRT registry and compared patients with CPA (+) to patients without CPA (-) to identify the risk factors for CPA during IHT.

### Transport of critically ill patients with RRT

RRT accompaniment refers to cases in which at least one RRT nurse and either the patient’s physician in charge or an RRT physician accompanied the traditional escort team. Cases accompanied by the RRT can be divided into two major categories: (1) If a patient in the general ward needs to be transported to the ICU or other room for diagnostic or therapeutic purposes and the transport risk is thought to be high, the intensivist of the RRT requests RRT accompaniment during transport; and (2) If a patient in the ICU needs to be transported for diagnostic or therapeutic purposes and the transport risk is thought to be high, the intensivist in charge of the ICU(who is also a member of the RRT) requests RRT accompaniment for the transport. The transport risk is subjectively determined by the intensivist, and RRT accompaniment in our hospital was limited to cases for which the RRT intensivist had requested RRT transportation support.

Before the launch of the RRT, critically ill patients were typically escorted by couriers and occasionally one or two physicians (usually an intern). Because in-hospital guidelines for IHT of critically ill patients have not been established in our institution, the transportation of high-risk patients was conducted inconsistently. Also, an escort decision, such as whether the patient would be accompanied by a physician, was dependent on the physician on duty (usually a trainee).

### Data collection

The following data were obtained from the RRT registry: patient demographic data, primary diagnosis, transport time, departure place, arrival place, airway and oxygen supply during transport, medications, CPA during transport, length of stay, and survival.

### Statistical analysis

Categorical variables and continuous variables are expressed as numbers and percentages, and mean ± standard deviation, respectively. Differences between the CPA (+) group and CPA (-) group were analyzed by the independent samples t-test for continuous variables and the chi-square test for categorical variables. A value of p < 0.05 was considered significant. Risk factors for AE during IHT were tested first by uni-variate analysis. Results were reported as odds ratios (OR), and statistical significance was ascertained by the 95% confidence interval. All statistical analyses were performed with SPSS version version 17.0 (SPSS Inc, Chicago, IL, USA).

### Ethics statement

The Institutional Review Board of Seoul National University Bundang Hospital approved the study protocol and waived the need for informed consent because of the retrospective study design (IRB number: B-1408-262-114).

## Results

**Fig 1:**
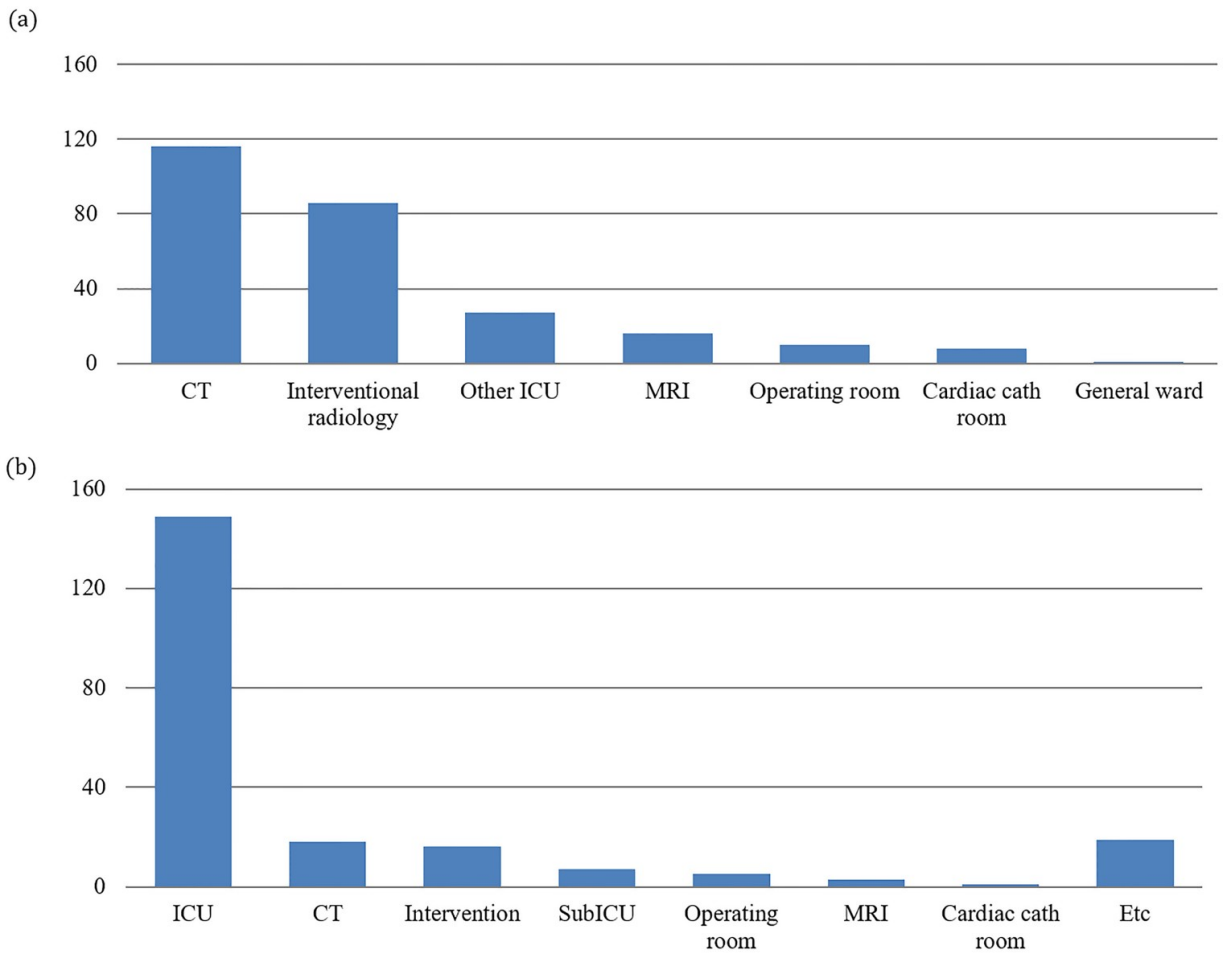
Transport origin and destination. (a) Transport destination from ICU departure, (b) Transport destination from general ward departure.

During the study period of 40 months, 558 critically ill patients were transported with the RRT. Of these, data from the following sets of patients were excluded: 11 patients due to the lack of information, seven patients who were admitted despite having no CPA event and five patients who were under 18 years of age. A final total of 535 patients were included in this study, of which eight (1.5%) developed CPA. Patients’ baseline demographics are described in Table 1. There were no significant differences in age, sex, and comorbidities between the CPA (+) and CPA (-) groups, but there were more patients with previous myocardial infarction (MI) in the CPA (+) group. ICU survival was significantly higher in the CPA (-) group than in the CPA (+) group (67.2% vs. 25.0%, p = 0.015). Origins of departure and transport destinations for included patients are depicted in Fig 1. Computed tomography rooms were the most frequent destination of ICU departures, while the ICU was the most frequent destination of ward departures.

**Table 1:** Characteristics of intra-hospital transport.

Variables	Total N = 535	CPA(+) N = 8	CPA(-) N = 527	P Value
**Age**	65.0(14.8)	52.1(20.5)	65.2(14.6)	0.056
**Male**	349(65.2)	4(50.0)	345(65.5)	0.362
**Charlson Comorbidity Index**	5.7(2.7)	4.4(2.5)	5.7(2.7)	0.131
**Underlying disease**				
Tumor	195(36.4)	2(25.0)	193(36.1)	0.498
Diabetes	124(23.2)	1(12.5)	123(23.3)	0.471
Chronic lung disease	108(20.2)	2(25.0)	106(20.1)	0.733
Cerebrovascular disease	100(18.7)	1(12.5)	99(18.8)	0.651
Chronic renal disease	60(11.2)	0	60(11.4)	0.311
Chronic liver disease	45(8.4)	1(12.5)	44(8.3)	0.675
Myocardial infarction	31(5.8)	3(37.5)	28(5.3)	<0.001
Peripheral vascular disease	5(0.9)	0	5(0.9)	0.782
**Department**				
Internal Medicine	269(50.3)	6(75.0)	263(49.9)	0.159
General Surgery	109(20.4)	0(0.0)	109(20.7)	0.149
Thoracic Surgery	56(10.5)	1(12.5)	55(10.4)	0.850
Neurology	23(4.3)	0	23(4.4)	0.546
Emergency room	18(3.4)	0	18(3.4)	0.595
Rehabilitation Medicine	11(2.1)	1(12.5)	10(1.9)	0.136
Others^a^	49(9.2)	0	49(9.2)	0.366
**Characteristics of hospitalization**				
Hospital survival	305(57.0)	2(25.0)	303(57.5)	0.065
ICU Survival	356(66.5)	2(25.0)	354(67.2)	0.012
Length of ICU stay (days)	17.6(31.5)	5.4(3.9)	17.7(31.7)	<0.001
Length of Hospital stay (days)	48.9(44.4)	26.9(33.5)	49.2(44.4)	0.032

Values are shown as number (percentage) or mean (standard deviation).

^a^Others included orthopedics (11), neurosurgery (9), otolaryngology (7), obstetrics and gynecology (7), spinal centers (7), urology (7), and plastic surgery (1).

Abbreviations: CPA, Cardio Pulmonary Arrest; ICU, Intensive Care Unit; SD, Standard Deviation.

Airway and oxygen supply received by patients during transport are described in Table 2. The mean fraction of inspired oxygen (FiO_2_) of patients receiving oxygen during transport was 69.8%, which was significantly higher in the CPA (+) group than in the CPA (-) group (100% vs. 69.3%, p = 0.001). A higher proportion of patients in the CPA (+) group than in the CPA (-) group received manual ventilation during transport (75.0% vs. 23.0%, p = 0.001).

**Table 2:** Oxygenation method and type of airway of included patients.

Variables	Total	CPA(+)	CPA(-)	P
	N = 535	N = 8	N = 527	Value
**Transport time (min)**	37.2(35.8)	14.8(11.7)	37.5(35.9)	0.045
**Required Fio2 (%)**	69.8(29.2)	100(0.0)	69.3(29.2)	0.001
**Oxygenation**				
Portable ventilator	251(46.9)	2(25.0)	249(47.2)	0.211
Manual ventilation using a bag-valve mask	127(23.7)	6(75.0)	121(23.0)	0.001
Facial Mask	61(11.6)	0	61(11.6)	0.307
Nasal prong	37(6.9)	0	37(7.0)	0.437
High Flow Nasal Cannula	25(4.7)	0	25(4.7)	0.528
Room air	25(4.7)	0	25(4.7)	0.528
T-piece	7(1.3)	0	7(1.3)	0.743
Home ventilator	2(0.4)	0	2(0.4)	0.861
**Type of airway**				
Artificial airway	381(71.2)	8(100)	373(70.8)	0.070
Endotracheal tube	330(61.7)	7(87.5)	323(61.3)	0.130
Tracheostomy	51(9.5)	1(12.5)	50(9.5)	0.773
No artificial airway	154(28.8)	0	154(29.2)	0.070
**Mode of Portable ventilator**				0.132
PCV	123(23.0)	2(25.0)	121(23.0)	
PSV	66(12.3)	0	66(12.5)	
SIMV	57(10.7)	0	57(10.8)	
VCV	3(0.6)	0	3(0.6)	
CPAP	2(0.4)	0	2(0.4)	
**Mode of ECMO**				0.595
VV	12(2.2)	0	12(2.3)	
VA	6(1.1)	0	6(1.1)	

Values are shown as number (percentage) or mean (standard deviation), or median (interquartile range). Abbreviations; CPA, Cardio Pulmonary Arrest; Fio2, Fraction of inspired oxygen; PCV, Pressure Control Ventilation; PSV, Pressure Support Ventilation; SIMV, Synchronized Intermittent Mechanical Ventilation; VCV, Volume Control Ventilation; CPAP, Continuous Positive Airway Pressure; ECMO, Extracorporeal Membrane Oxygenation; VV, Veno Venous; VA, Veno Arterial.

A total of 239 (44.7%) patients received continuous vasopressor infusion, and the most commonly used vasopressor was norepinephrine, with 217 (40.2%) patients receiving a mean amount of 19.8 (±21.9) mcg/min (Table 3). There was a significant difference in the number of vasopressors received between the CPA (+) and CPA (-) groups; the greater the number of vasopressors used, the higher the risk of CPA (p<0.001). In addition, 171 (32.0%) patients received continuous infusion of a sedative, with remifentanyl and dexmedetomidine being the most commonly used. No difference in the type and the dose of sedative was found between patients with or without CPA during IHT.

**Table 3:** Use of sedatives and vasopressors during intra-hospital transport.

Variables	Total	CPA(+)	CPA(-)	P	Mean dose	Unit
	N = 535	N = 8	N = 527	Value		
**Continuous vasopressors**	239(44.7)	6(75.0)	233(44.2)	0.082		
Norepinephrine	215(40.2)	6(75.0)	209(39.1)	0.043	19.8(±21.9)	mcg/min
Dopamine	59(11.0)	3(37.5)	56(10.6)	0.016	13.1(±11.6)	mcg/kg/min
Dobutamine	26(4.9)	2(25.0)	24(4.6)	0.008	11.2(±8.7)	mcg/kg/min
Epinephrine	27(5.0)	2(25.0)	25(4.7)	0.009	0.09(±0.05)	mcg/kg/min
Vasopressin	26(4.9)	1(12.5)	25(4.7)	0.311	0.06(±0.05)	unit/min
**Number of vasopressor**				< 0.001		
0 Vasopressor	296(55.3)	2(25.0)	294(55.8)			
1 Vasopressor	167(31.2)	3(37.5)	164(31.1)			
2 Vasopressors	42(7.9)	0(0.0)	42(8.0)			
3 Vasopressors	19(3.6)	2(25.0)	17(3.2)			
4 Vasopressors	10(1.9)	0(0.0)	10(1.9)			
5 Vasopressors	1(0.2)	1(12.5))	0(0.0)			
**Continuous sedatives**	171(32.0)	1(12.5)	170(32.2)	0.234		
Remifentanyl	110(20.6)	1(12.5)	109(20.7)	0.570	0.08(±0.06)	mcg/kg/min
Dexmedetomidine	96(17.9)	0(0.0)	96(18.2)	0.183	0.49((±0.35)	mcg/kg/hr
Midazolam	52(9.7)	0(0.0)	52(9.9)	0.350	3.96((±1.9)	mg/min
Cisatracurium	43(8.0)	0(0.0)	43(8.2)	0.400	2.45((±1.3)	mcg/kg/min
Vecuronium	8(1.5)	0(0.0)	8(1.5)	0.725	5.21((±1.4)	mg/min
**Number of sedatives**				0.735		
0 Sedative	364(68.0)	7(87.5)	357(67.7)			
1 Sedative	69(12.9)	1(12.5)	68(12.9)			
2 Sedatives	64(12.0)	0(0.0)	64(12.1)			
3 Sedatives	34(6.4)	0(0.0)	34(6.5)			
4 Sedatives	4(0.7)	0(0.0)	4(0.8)			
5 Sedatives	0(0.0)	0(0.0)	0(0.0)			

Values are shown as number (percentage) or mean ± standard deviation. Abbreviations: CPA, Cardio Pulmonary Arrest.

We examined which parameters could predict CPA during IHT. In the univariate analysis, age (OR = 0.9 [0.90–0.99]; p = 0.018), APACHE-II score (OR = 1.1 [1.02–1.19]; p = 0.012), history of myocardial infarction (OR = 10.7 [2.4–47.0]; p = 0.002), manual ventilation using a bag-valve mask (OR = 10.1 [2.0–50.5], p = 0.005), and the use of three or more vasopressors (OR = 11.1 [2.5–48.9], p = 0.001) were significant (Table 4). Our result showed that the lower the age, the higher the risk of CPA but this seems to be due to the small number of CPA (n = 8) patients including those with younger age.

**Table 4:** Univariate analysis of risk factors for cardiopulmonary arrest during intra-hospital transport.

Variables	Odd ratio	95% CI	P value
Age	0.9	0.90–0.99	0.018
Sex, male	0.5	0.10–2.10	0.370
APACHE-II score	1.1	1.02–1.19	0.012
Charlson Comorbidity Index	0.8	0.60–1.10	0.173
History of myocardial infarction	10.7	2.40–47.0	0.002
Manual ventilation using a bag-valve mask	10.1	2.0–50.5	0.005
Required FiO_2_	1.0	0.9–1.0	0.053
Three or more vasopressors	11.1	2.5–48.9	0.001

Abbreviations: APACHE, Acute Physiology and Chronic Health Evaluation; Fio2, Fraction of Inspired Oxygen; CI, Confidence Interval

The characteristics of each of the eight CPA cases are briefly summarized in Table 5. Among these cases, manual ventilation with mask bag valve was used in six cases while only two cases used portable ventilators. Required FiO_2_ for all was 100%.

**Table 5:** Characteristics of the eight patients with cardiopulmonary arrest during intrahospital transport with the rapid response team.

Case	Sex	Age	Departure	Destination	MI	Manual ventilation	Portable ventilator	Three or more vasopressors	Initial rhythm	Airway	FiO_2_	Survival	Type of arrest	Diagnosis
1	M	44	CCU	ICU	Y		Y	Y	Ventricular tachycardia	E	100	S	C	STEMI
2	F	77	Ward	ICU	Y	Y			Asystole	E	100	D	R	Sepsis, OM
3	F	57	Ward	ICU		Y		Y	Asystole	E	100	D	R	Sepsis, SICMP
4	M	21	ICU	CT room			Y		PEA	E	100	D	R	HFRS,ICH
5	F	57	ICU	CT room	Y	Y		Y	Asystole	E	100	D	C	3VD, Acute stroke
6	F	25	Ward	ICU		Y			Asystole	E	100	D	C	Pulmonary embolism
7	M	70	Ward	ICU		Y			Asystole	E	100	D	R	Lung cancer MPE
8	M	66	Ward	Interventional radiology		Y			Asystole	T	100	S	R	Tetraplegia Pneumonia

Abbreviations: ICU, intensive care unit; MI, myocardial infarction; CPA, cardiopulmonary arrest; OM, osteomyelitis; E tube, endotracheal tube; T-tube, tracheal tube; STEMI, ST elevated myocardial infarction; SICMP, stress induced cardiomyopathy; PEA, Pulseless electrical activity; HFRS, Hantavirus hemorrhagic fever with renal syndrome; ICH, intracerebral hemorrhage; 3VD, three vessel disease; MPE, Malignant pleural effusion; S, survaval; D, death; FiO_2_, Fraction of inspired oxygen; C, Cardiac arrest; R, Respiratory arrest

### Subgroup analysis

We limited the comparison to patients with similar levels of critical illness to find what factors are associated with safer transport for very unstable patients because oftentimes, very unstable patients still need transport. We compared the risk factors of CPA in a subgroup of patients with advanced airway, at least one vasopressor, requiring an advanced airway, and three vasopressors (S1–S3 Tables).

In the first subgroup (patients with advanced airway, N = 381), eight cases of CPA (2.1%) occurred. Differences in patients’ characteristics and important variables are described in S1 Table. We found similar risk factors for CPA during IHT among this population compared with the original full population. Age and APACHE-II score were also significant, but each OR was closer to 1.0. Especially in this group, the use of portable ventilator significantly reduced the CPA risk during IHT (OR = 0.17 [0.03–0.83], p = 0.029). Also, we found that the risk increased significantly when the ward was the departure point and the ICU was the destination.

In the second subgroup (patients with vasopressor support, N = 239), six patients (2.5%) had CPA during RRT-led IHT (S2 Table). Overall, similar risk factors were observed, but history of hemiplegia was also significant (OR = 9.5 [1.19–76.29], p = 0.033) in this group.

In the third subgroup (patients with advanced airway and more than three vasopressors, N = 28), three patients (10.7%) had CPA (S3 Table). Because of the small number of the patients in this subgroup (N = 28), only history of myocardial infarction was observed to be marginally significant (OR = 10.7 [0.92–124.38], p = 0.058).

## Discussion

In this study, we aimed to determine the occurrence rate of CPA during IHT among patients accompanied by the RRT, and to identify risk factors for CPA during transport. We determined that 1.5% of critically ill patients transported with an RRT developed CPA during transport, and that history of MI, manual ventilation with a bag-valve mask, and the use of three or more vasopressors were independent risk factors for developing CPA during transport. Age and APACHE-II score appeared to be significant also, but each OR was near 1.0, and there were two young patients in their 20’s among the eight CPA patients (Table 5). Thus the OR value, which indicated that the risk of CPA increased as the age decreased, should be interpreted along with other clinical findings.

Critically ill patients require various diagnostic imaging and interventions due to the complexities of their medical needs, and IHT for these patients is often inevitable. When clinicians make the decision to transport these patients within a hospital, it is essential to consider whether the intervention that requires transport will benefit the patient; considering possible risks that can arise during transit is equally important [2, 10]. To estimate the risk-benefit of transport, it would be helpful to identify patients with a high risk for the development of complications during or after transport.

Suggested risk factors of AEs during IHT in previous research include the type of transport (emergency transports in particular) [8, 9], the number of infusion pumps [11] especially with catecholamine [8, 12], positive end expiratory pressure [6, 8, 13], and sedation before transport [13]. However, the definition of AEs varied in each of these studies, and Fanara et al. [14] mentioned that most of the risk factors described in these studies did not have any significant statistical value and were usually based on the good clinical sense of the authors.

The four main categories of possible risk factors for AEs during IHT [14] comprise equipment factors, human factors, organizational factors, and patient factors (i.e. clinical instability). Although patient-related risk factors are difficult to identify, other risk factors such as equipment-related, human error, and organizational factors might be controlled more easily [14]. An RRT can address most of the risk categories described above. The four basic elements of IHT guidelines cover communication, qualified personnel, proper equipment, and monitoring [1]. Since the RRT is comprised of staffs who are competent and skilled in caring for critically ill patients, the RRT can generally fulfill these recommendations in institutions, such as ours, where a specialized dedicated transport team has not been formed due to workforce limitations. RRT staffs are already specialized for care of critically ill patients, and most RRT have their own equipment, emergency medicines, and monitoring devices needed for critical situations. An RRT also has the advantage of being able to contact a team leader (intensivist) directly in the event of a serious problem during transfer, so that the chain of action can be very fast in terms of communication and problem solving.

Considering the role that the RRT filled in our institution, we aimed to understand the rates of CPA occurrence and risk factors for CPA in critically ill patients who were transported by this highly trained team. We found that the rate of CPA in this patient population in our institution (1.5%) was similar to that in previous studies (ranging from 0.3–3%) [5–9]. Although this may be considered a low rate, the consequences can be so devastating that some might consider it an event that should “never” occur [3]. Thus, it is essential to understand the risk factors underlying the occurrence of CPA in critically ill patients transported by an RRT.

In our study, history of MI was found to be a significant risk factor for CPA. Minor hemodynamic changes in blood pressure, electrocardiogram rhythm, and heart rate (acceleration and deceleration) can result in a major series of events that put vulnerable patients at a high risk of severe outcomes. In a study by Taylor et al. [15], arrhythmia occurred during transport in 85% of 55 patients with cardiac disease. It is well known that risk of sudden cardiac death increases after MI, with an overall incidence ranging from 2–4% per year in many large observational and randomized studies [16–20]. Therefore, the results of our study are in line with previous results, and show that patients with a history of MI are particularly vulnerable to CPA during IHT, thus requiring additional attention and care.

The second risk factor for developing CPA during IHT when accompanied by RRT was use of three or more vasopressors. In a study by Parmentier-Decrucq et al. [13], increased risk of AE was significantly associated with fluid challenge during transport (OR 6.5, 95% CI 1.3–31.7; p = 0.021). Additionally, the number of infusion pumps [11], especially with catecholamine [8, 12] were suggested as risk factors for AE during IHT. However, there is no available evidence establishing the upper limits of number or dose of each vasopressor that can be safely permitted during patient transport. In the Mayo Clinic’s IHT protocol [2], the most at-risk patients are classified as Level 4 transports, which require advanced medical care and monitoring. One of the defining criteria for Level 4 transport is “continued manipulation of vasoactive infusions”, which is unclear and ambiguous. Although our study found that the risk of CPA increased by more than 10-fold (OR 11.1, 95% CI 2.548.9, p = 0.001) when three or more vasopressors were used during IHT, we could not determine the optimum cutoff level of vasopressor dose to contraindicate IHT. Therefore, the amount of vasopressor for transport contraindication remains to be established.

Manual ventilation was found to be a significant risk factor for CPA in our study. Manual ventilation has been widely used due to its simple manipulation and good portability. In a single blind prospective study [21], manual ventilation during IHT of mechanically ventilated critically ill patients was determined to be safe when provided by trained personnel (e.g. a respiratory therapist). However, there are also concerns about the potential risk of hyperventilation [22] or inconsistent respiration [23]. Braman et al. [24] identified that mean changes in PCO2 and pH were significantly lower in a portable mechanical ventilator group than in a manual ventilatory group (p<0.01). Although previous studies have shown that manual ventilation induces changes in physiologic variables or in arterial blood gases, these studies only mentioned as a matter of concern that manual ventilation could lead to fatal AEs [22–25]. Our study is meaningful in that it shows that the risks of CPA during IHT are significantly increased with the use of manual ventilation OR 10.1, 95% CI 2.0–50.5, p = 0.005). However, there are still no clear guidelines for determining the use of portable ventilators according to oxygen level or ventilator pressure. As individual institutions each have different resources, these thresholds should be determined to aid in effective resource distribution. In a transport protocol of the Mayo Clinic [2], use of a portable ventilator is recommended if the required FiO2 is greater than 40%, positive end-expiratory pressure >5 mmHg, and respiratory rate >20 bpm, but it also relies on expert opinion and experience, rather than the results from a well-designed study.

In our three subgroup analyses (S1, S2 and S3 Tables), relatively consistent significant risk factors of CPA during RRT-led IHT included, history of myocardial infarction, manual ventilation using a bag-valve mask, and the use of three or more vasopressors. Required FiO2 appeared to be less important. Especially in the subgroup with artificial airways (n = 381), using portable ventilator was found to significantly reduce the CPA risk during transport (OR = 0.166 [0.033–0.834], p = 0.029). Also, the risk increased significantly when the ward was the departure point and the ICU was the destination. It is thought that CPA occurs during IHT while the patient is being moved to the ICU without being fully stabilized in the ward room. Therefore, In this case, one needs to both be extremely careful and to move the patient to the ICU as soon as possible; however, it is also necessary to ensure maximum stabilization in the ward room as much as possible and before starting the transfer. History of hemiplegia was especially significant (OR = 9.54 [1.19–76.29], p = 0.033) in the subgroup with vasopressors (N = 239). The most common cause of hemiplegia is stroke; and both MI and stroke are the most important cerebrovascular diseases. Therefore, the ischemic episodes in cerebrovascular diseases would be significant risk factors for CPA during IHT especially in those with one or more vasopressors’ support.

Our study has several limitations. First, selection bias may have occurred as our data does not represent all transported patients who were in critical condition in the hospital in the study period. The RRT cannot accompany every transport of all critical patients due to limited human and equipment resource; thus, RRT accompaniment is limited to cases for which an intensivist requests RRT transport. This leads to the potential problem of high-risk patients being defined arbitrarily by the intensivist in the team, as described in the methods section. Secondly, although our findings showed that manual ventilation and number of vasopressors used are risk factors for CPA during IHT, our findings did not provide specific cutoffs for portable ventilator use, or specific vasopressor dosage limit during transport. Thirdly, we did not review minor variations in physiologic parameters as complications of transport. Because this study was a retrospective review, we focused on the definite outcome of CPA to reduce bias. Additionally, previous studies [5, 7, 8, 26, 27] have evaluated the outcome data of patients who were transported, and reported that AEs such as arterial blood gas changes, physiologic changes, and equipment-related problems are frequent. Fourth, we did not compare CPA rates before and after implementation of RRT accompanied transport to determine whether accompaniment by a specialized team such as the RRT can reduce AEs during IHT. There are only a few existing studies evaluating whether implementation of a dedicated specialized transport team for critically ill patients promotes stable transport and patient safety [2, 3, 10]. The level of evidence is weak, and none of these previous studies were well-designed prospective studies. Further prospective study is needed to provide evidence that implementation of a team trained specifically in the transfer of critically ill patients is cost-effective, that it reduces the occurrence of harmful side effects and accidents during transportation, and that it improves overall patient outcomes.

## Conclusions

This is the first study to report the incidence rate of CPA in critically ill patients during IHT accompanied by the RRT. The occurrence rate of CPA was 1.5%. Furthermore, we determined that history of MI, manual ventilation with a bag-valve mask, and the use of three or more vasopressors were independent risk factors of CPA during IHT of critically ill patients who were accompanied by the RRT. Despite the use of this type of specialized team, CPA still occurred during IHT, and special attention should be devoted to high-risk, critically ill patients, particularly those with the risk factors determined by this study. Further prospective studies with larger sample sizes are needed to clarify when and how a portable ventilator should be used in the transport of critically ill patients, and to confirm the cutoff point for number of vasopressor use during transport.

## Supporting information

S1 Table Patients with advanced airway. (DOCX)

S2 Table Patients with vasopressors. (DOCX)

S3 Table Patients with artificial airway and three or more vasopressors. (DOCX)
