# Supplementary material for: Intra-hospital transport of critically ill patients with rapid response team and risk factors for cardiopulmonary arrest: A retrospective cohort study
Source: PLoS One. 2019 Mar 5;14(3):e0213146. doi: 10.1371/journal.pone.0213146 (PMC6400377; doi:10.1371/journal.pone.0213146)
Supplement: S1 Table — (DOCX) [file pone.0213146.s001.docx]

**S1 Table. Patients with advanced airway**

| **Variables** | **Total** | **CPA(+)** | **CPA(-)** | **P** | **Univariate analysis** | | |
| --- | --- | --- | --- | --- | --- | --- | --- |
|  | **N= 381** | **N= 8** | **N= 373** | **Value** | **OR** | **95% CI** | **P-value** |
| **Age** | 64.6(15.0) | 51.4(22.1) | 64.8(14.8) | 0.019 | 1.0 | 0.91-0.99 | 0.022 |
| **Male** | 245(64.5) | 4(57.1) | 241(64.6) | 0.683 |  |  |  |
| **Charlson Comorbidity Index** | 5.6(2.7) | 4.4(2.5) | 5.6(2.7) | 0.163 |  |  |  |
| **APACHE-II score(valid:328)** | 1.073 | 37.1(8.6) | 31.24(9.3) | 0.032 | 1.1 | 1.01-1.19 | 0.023 |
| **Underlying disease** |  |  |  |  |  |  |  |
| Hemiplegia | 10(2.6) | 1(14.3) | 9(2.4) | 0.052 | 6.7 | 0.73-61.93 | 0.092 |
| Chronic renal disease | 46(12.1) | 0(0.0) | 46(12.3) | 0.322 |  |  |  |
| Chronic liver disease | 36(9.5) | 1(14.3) | 35(9.4) | 0.661 |  |  |  |
| Myocardial infarction | 22(5.8) | 3(37.5) | 19(5.1) | <0.001 | 11.2 | 2.49-50.30 | 0.002 |
| Peripheral vascular disease | 3(0.8) | 0(0.0) | 3(0.8) | 0.812 |  |  |  |
| **Departure** |  |  |  |  |  |  |  |
| Ward | 101(26.5) | 5(62.5) | 96(25.7) | 0.020 | 4.8 | 1.13-20.50 | 0.034 |
| ICU | 241(63.3) | 3(37.5) | 238(63.8) | 0.127 |  |  |  |
| Emergency room | 23(6.0) | 0 | 23(6.2) | 0.469 |  |  |  |
| Operating room | 4(1.0) | 0 | 4(1.1) | 0.768 |  |  |  |
| Dialysis room | 2(0.5) | 0 | 2(0.5) | 0.835 |  |  |  |
| Others | 8(2.1) | 0 | 8(2.1) | 0.675 |  |  |  |
| **Arrive** |  |  |  |  |  |  |  |
| ICU | 136(35.7) | 6(75.0) | 130(34.9) | 0.019 | 5.6 | 1.12-28.18 | 0.036 |
| CT room | 136(35.7) | 2(25) | 134(35.9) | 0.523 |  |  |  |
| MRI room | 20(5.2) | 0 | 20(5.4) | 0.501 |  |  |  |
| Cardiac cath lab | 13(3.4) | 0 | 13(3.5) | 0.123 |  |  |  |
| Ward | 3(0.8) | 0 | 3(0.8) | 0.799 |  |  |  |
| Operating room | 5(1.3) | 0 | 5(1.3) | 0.742 |  |  |  |
| Sub-ICU | 2(0.5) | 0 | 2(0.5) | 0.835 |  |  |  |
| Others | 9(2.3) | 0 | 9(2.3) | 0.799 |  |  |  |
| **Required Fio2 (%)** | 72.7(27.6) | 100(0.0) | 72.2(27.6) | 0.004 | 1.1 | 0.99-1.15 | 0.097 |
| **Oxygenation** |  |  |  |  |  |  |  |
| Portable ventilator | 251(65.9) | 2(25.0) | 249(66.8) | 0.014 | 0.2 | 0.03-0.83 | 0.029 |
| Manual ventilation using a bag-valve mask | 121(31.8) | 6(75.0) | 115(30.8) | 0.008 | 4.9 | 1.09-22.31 | 0.039 |
| T-piece | 7(1.8) | 0(0.0) | 7(1.9) | 0.714 |  |  |  |
| Home ventilator | 2(0.5) | 0(0.0) | 2(0.5) | 0.846 |  |  |  |
| **Type of airway** |  |  |  |  |  |  |  |
| Endotracheal tube | 330(86.6) | 7(87.5) | 323(86.6) | 0.941 |  |  |  |
| Tracheostomy | 51(13.4) | 1(12.5) | 50(13.4) | 0.941 |  |  |  |
| **Continuous vasopressors** | 201(52.8) | 6(75.0) | 195(52.3) | 0.203 |  |  |  |
| Norepinephrine | 181(47.5) | 6(75.0) | 175(46.9) | 0.116 |  |  |  |
| Dopamine | 51(13.4) | 3(37.5) | 48(12.9) | 0.043 |  |  |  |
| Dobutamine | 25(6.6) | 2(25.0) | 23(6.2) | 0.033 |  |  |  |
| Epinephrine | 24(6.3) | 2(25.0) | 22(5.9) | 0.028 |  |  |  |
| Vasopressin | 19(5.0) | 0(0.0) | 19(5.1) | 0.540 |  |  |  |
| **Number of vasopressor** |  |  |  | <0.001 |  |  |  |
| 0 Vasopressor | 180(47.2) | 2(25.0) | 178(47.7) |  | 1.0 |  |  |
| 1 Vasopressor | 135(35.4) | 3(37.5) | 132(35.4) |  | 1.9 | 0.37-9.71 | 0.448 |
| 2 Vasopressors | 38(10.0) | 0(0.0) | 38(10.2) |  | 0.9 | 0.04-19.70 | 0.961 |
| 3 Vasopressors | 18(4.7) | 2(25.0) | 16(4.3) |  | 10.1 | 1.47-84.34 | 0.020 |
| 4 Vasopressors | 9(2.4) | 0(0.0) | 9(2.4) |  | 3.8 | 0.17-83.89 | 0.403 |
| 5 Vasopressors | 1(0.3) | 1(12.5) | 0 |  |  |  |  |
| **Three or more vasopressor** | 28(7.3) | 3(37.5) | 25(6.7) | 0.001 | 8.7 | 2.11-35.29 | 0.003 |
| **Continuous sedatives** | 170(44.7) | 1(14.3) | 169(45.3) | 0.065 |  |  |  |
| Remifentanyl | 109(28.6) | 1(12.5) | 108(29.0) | 0.308 |  |  |  |
| Dexmedetomidine | 95(24.9) | 0(0.0) | 95(25.5) | 0.099 |  |  |  |
| Midazolam | 52(13.6) | 0(0.0) | 52(13.9) | 0.256 |  |  |  |
| Cisatracurim | 42(11.0) | 0(0.0) | 42(11.3) | 0.314 |  |  |  |
| Vecuronium | 8(2.1) | 0(0.0) | 8(2.1) | 0.675 |  |  |  |
| **Number of sedatives** |  |  |  | 0.429 |  |  |  |
| 0 Sedative | 211(55.4) | 7(85.7) | 204(54.7) |  |  |  |  |
| 1 Sedative | 69(18.1) | 1(12.5) | 68(18.2) |  |  |  |  |
| 2 Sedatives | 64(16.8) | 0(0.0) | 64(17.2) |  |  |  |  |
| 3 Sedatives | 33(8.7) | 0(0.0) | 33(8.8) |  |  |  |  |
| 4 Sedatives | 4(1.0) | 0(0.0) | 4(1.1) |  |  |  |  |

Values are shown as number (percentage) or mean (standard deviation)

Abbreviations; CPA, Cardio Pulmonary Arrest; OR, Odds ratio; CI, Confidence interval; **APACHE,** Acute Physiology and Chronic Health Evaluation; ICU, Intensive care unit; CT, Computed tomography; MRI, Magnetic resonance imaging; Cardiac cath lab, Cardiac catheterization laboratory; Fio2, Fraction of inspired oxygen.
