# Supplementary material for: Intra-hospital transport of critically ill patients with rapid response team and risk factors for cardiopulmonary arrest: A retrospective cohort study
Source: PLoS One. 2019 Mar 5;14(3):e0213146. doi: 10.1371/journal.pone.0213146 (PMC6400377; doi:10.1371/journal.pone.0213146)
Supplement: S2 Table — (DOCX) [file pone.0213146.s002.docx]

**S2 Table. Patients with vasopressors**

| Variables | Total | CPA(+) | CPA(-) | P | Univariate analysis | | |
| --- | --- | --- | --- | --- | --- | --- | --- |
|  | N= 239 | N= 6 | N= 233 | Value | OR | 95% CI | P-value |
| Age | 64.4(13.2) | 52.5(17.8) | 64.7(12.9) | 0.070 | 0.9 | 0.89-0.99 | 0.026 |
| Male | 146(61.1) | 4(66.7) | 142(60.9) | 0.777 |  |  |  |
| Charlson Comorbidity Index | 5.4(2.8) | 4.3(1.5) | 5.8(2.8) | 0.168 | 0.8 | 0.61-1.13 | 0.235 |
| APACHE-II score(valid:213) | 32.7(10.1) | 41.8(9.0) | 32.4(9.9) | 0.019 | 1.1 | 1.01-1.22 | 0.025 |
| Underlying disease |  |  |  |  |  |  |  |
| Hemiplegia | 7(2.9) | 1(16.7) | 6(2.6) | 0.043 | 9.5 | 1.19-76.29 | 0.033 |
| Myocardial infarction | 15(6.3) | 2(33.3) | 13(5.6) | 0.006 | 9.1 | 1.69-48.62 | 0.010 |
| Duration of transport | 43.8(35.3) | 18.3(11.4) | 44.4(35.5) | 0.043 |  |  |  |
| Departure |  |  |  |  |  |  |  |
| Ward | 63(26.4) | 3(50.0) | 60(25.8) | 0.183 | 2.9 | 0.63-13.09 | 0.174 |
| ICU | 160(66.9) | 157(67.4) | 3(50.0) | 0.371 | 0.5 | 0.11-2.21 | 0.350 |
| Arrive |  |  |  |  |  |  |  |
| Ward | 76(31.8) | 4(66.7) | 72(30.9) | 0.063 |  |  |  |
| ICU | 2(0.8) | 0 | 2(0.9) | 0.820 | 4.0 | 0.83-19.42 | 0.084 |
| Survival | 101(42.3) | 2(33.3) | 99(42.5) | 0.654 |  |  |  |
| Required Fio2 (%) | 71.6(28.5) | 100 | 70.9(28.5) | 0.009 | 1.1 | 0.99-1.14 | 0.088 |
| Way of Oxygen inhalation |  |  |  |  |  |  |  |
| Portable ventilator | 140(58.6) | 2(33.3) | 138(59.2) | 0.204 | 0.4 | 0.08-1.85 | 0.232 |
| Manual ventilation using a bag-valve mask | 57(23.8) | 4(66.7) | 53(22.7) | 0.013 | 6.1 | 1.25-29.59 | 0.026 |
| T-piece |  |  |  |  |  |  |  |
| Home ventilator |  |  |  |  |  |  |  |
| Type of airway |  |  |  |  |  |  |  |
| Artificial airway | 201(84.1) | 6(100) | 195(83.7) | 0.281 | 2.6 | 0.17-48.12 | 0.530 |
| Endotracheal tube |  |  |  |  |  |  |  |
| Tracheostomy |  |  |  |  |  |  |  |
| Continuous vasopressors |  |  |  |  |  |  |  |
| Norepinephrine | 215(90.0) | 6(100) | 209(89.7) | 0.407 |  |  |  |
| Dopamine | 59(24.7) | 3(50.0) | 56(24.0) | 0.145 | 3.1 | 0.69-14.37 | 0.140 |
| Dobutamine | 26(10.9) | 2(33.3) | 24(10.3) | 0.074 | 4.8 | 0.94-24.08 | 0.060 |
| Epinephrine | 27(11.3) | 2(33.3) | 25(10.7) | 0.084 | 4.5 | 0.89-22.97 | 0.067 |
| Vasopressin |  |  |  |  |  |  |  |
| Number of vasopressor |  |  |  | <0.001 |  |  |  |
| 1 Vasopressor | 167(69.9) | 3(50.0) | 164(70.4) |  |  |  |  |
| 2 Vasopressors | 42(17.6) | 0 | 42(18.0) |  |  |  |  |
| 3 Vasopressors | 19(7.9) | 2(33.3) | 17(7.3) |  |  |  |  |
| 4 Vasopressors | 10(4.2) | 0 | 10(4.3) |  |  |  |  |
| 5 Vasopressors | 1(4.2) | 1(16.7) | 0 |  |  |  |  |
| Three or more vasopressor | 30(12.6) | 3(50) | 27(11.6) | 0.005 | 7.5 | 1.60-35.35 | 0.011 |

Values are shown as number (percentage) or mean (standard deviation).

Abbreviations; CPA, Cardio Pulmonary Arrest; OR, Odds ratio; CI, Confidence interval; APACHE**,** Acute Physiology and Chronic Health Evaluation; ICU, Intensive care unit; Fio2, Fraction of inspired oxygen.
