# Supplementary material for: Intra-hospital transport of critically ill patients with rapid response team and risk factors for cardiopulmonary arrest: A retrospective cohort study
Source: PLoS One. 2019 Mar 5;14(3):e0213146. doi: 10.1371/journal.pone.0213146 (PMC6400377; doi:10.1371/journal.pone.0213146)
Supplement: S3 Table — (DOCX) [file pone.0213146.s003.docx]

**S3 Table. Patients with artificial airway and 3 or more vasopressors (N=28)**

| Variables | Total | CPA(+) | CPA(-) | P | Univariate analysis | | | |  |
| --- | --- | --- | --- | --- | --- | --- | --- | --- | --- |
|  | N= 28 | N= 3 | N= 25 | Value | OR | 95% CI | P-value | |  |
| Age | 66.5(12.8) | 52.7(7.5) | 68.2(12.3) | 0.051 | 0.9 | 0.82-1.02 | | 0.102 | |
| Male | 15(53.6) | 1(33.3) | 14(56.0) | 0.457 |  |  | |  | |
| Charlson Comorbidity Index | 5.0(2.7) | 3(1) | 5.24(2.80) | 0.28 |  |  | |  | |
| APACHE-II score(valid:24) | 33.9(10.5) | 38.7(13.0) | 33.2(10.3) | 0.505 | 1.0 | 0.93-1.17 | | 0.488 | |
| Underlying disease |  |  |  |  |  |  | |  | |
| Hemiplegia | 1(3.6) | 0 | 1(4.0) | 0.724 |  |  | |  | |
| Myocardial infarction | 5(17.9) | 2(66.7) | 3(12.0) | 0.019 | 10.7 | 0.92-124.38 | | 0.058 | |
| Peripheral vascular disease |  |  |  |  |  |  | |  | |
| Duration of transport | 32.8(23.1) | 12.3(13.6) | 35.2(23.0) | 0.09 |  |  | |  | |
| Departure |  |  |  |  |  |  | |  | |
| Ward | 1(3.6) | 1(33.3) | 0 | 0.003 |  |  | |  | |
| ICU | 26(92.9) | 2(66.7) | 24(96.0) | 0.062 |  |  | |  | |
| Arrive |  |  |  |  |  |  | |  | |
| Ward | 0 |  |  |  |  |  | |  | |
| ICU | 7(25.0) | 2(66.7) | 5(20.0) | 0.078 |  |  | |  | |
| LOS, hospital | 30.0(28.2) | 29.7(37.3) | 30.0(27.7) | 0.999 |  |  | |  | |
| LOS, ICU | 19.4(24.0) | 4.7(3.5) | 21.1(24.8) | 0.314 |  |  | |  | |
| Survival | 8(28.6) | 1(33.3) | 7(28.0) | 0.847 |  |  | |  | |
| Required Fio2 (%) | 86.6(22.1) | 100 | 85.04(22.91) | 0.28 | 1.0 | 0.95-1.10 | | 0.506 | |
| Way of Oxygen inhalation |  |  |  |  |  |  | |  | |
| Portable ventilator | 16(57.1) | 1(33.3) | 15(60.0) | 0.378 |  |  | |  | |
| Manual ventilation using a bag-valve mask | 11(39.3) | 2(66.7) | 9(36.0) | 0.304 | 2.9 | 0.95-1.10 | | 0506 | |
| Artificial airway | 28(100) | 3(100) | 25(100) |  |  |  | |  | |
| Number of vasopressor |  |  |  | 0.009 |  |  | |  | |
| 3 Vasopressors | 18(64.3) | 2(66.7) | 16(64.0) |  |  |  | |  | |
| 4 Vasopressors | 9(32.1) | 0 | 9(36.0) |  |  |  | |  | |
| 5 Vasopressors | 1(3.6) | 1(33.3) | 0 |  |  |  | |  | |

Values are shown as number (percentage) or mean (standard deviation).

Abbreviations; CPA, Cardio Pulmonary Arrest; OR, Odds ratio; CI, Confidence interval; APACHE, Acute Physiology and Chronic Health Evaluation; ICU, Intensive care unit; LOS, Length of stay; Fio2, Fraction of inspired oxygen.
